# Prevention is Better than Cure: A Hands-On, Play-Based, Innovative, Health and Well-Being Program in Remote Australia

**DOI:** 10.3390/children1030318

**Published:** 2014-10-16

**Authors:** Lis Mathiasen

**Affiliations:** Association for the Welfare of Children in Hospital WA Inc., Princess Margaret Hospital for Children, BOX D184, Perth 6840, Western Australia, Australia; E-Mail: lismat@iinet.net.au; Tel.: +61-8-93072148

**Keywords:** prevention, empowerment, physical health, mental health, play

## Abstract

A key to improving the quality of life in remote communities is the empowerment of children who are at health and educational risk. Between 2002 and 2009, at a remote Aboriginal school, students and community members participated in an innovative, play-based health and well-being program aimed at helping children to become self-determining and responsible for their own health and well-being. Holistic in its approach, and broad in its scope, the multi-faceted program encompassed the fundamentals of personal hygiene; understanding of body systems; the importance of nutrition, hydration, sleep and exercise; brain care; the biology of emotions, with particular emphasis on anger management and the critical interplay between emotions and behavior; the impact of substances of abuse on the brain; as well as the Hospital Familiarization Program (HFP) which prepares children for planned and unplanned hospitalization. Program outcomes included improved school attendance and student engagement; increased community awareness of the importance of a healthy lifestyle; improved self-concept, self-esteem and self-confidence; as well as increased respect and caring for self and others. A reduction in children’s fear and anxiety when facing hospitalization and visits to the doctor was also evident. Each year, 12,500 children throughout Western Australia enjoy the benefits of the HFP.

## 1. Introduction

This paper describes the rationale, theoretical underpinnings and the process of the implementation of a play based, hands on health and well-being program in a remote community. The program was three-fold, featuring the key elements of physical health, social and emotional well-being and a hospital familiarization program that featured role-play as an important component.

**Figure 1:**
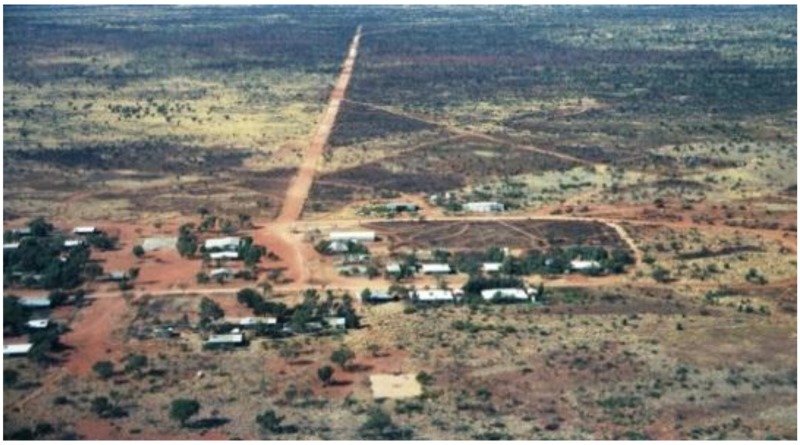
Ngaanyatjarra Lands

The article is based on my experience as a teacher and principal, working in a small Aboriginal community school in the Ngaanyatjarra Lands, over a period of eight years (2002–2009). Approximately, 150 students aged from five to 16 years attended the school over this period of time. Located in the Gibson Desert, 2000 km north east of Perth, the Ngaanyatjarra Lands is home to a cluster of the most isolated communities in Western Australia. The community is situated approximately 30 km west of the Northern Territory border and lies to the south east of Lake Hopkins on the Sandy Blight Road, see Figure 1. The nearest major town in Western Australia is Kalgoorlie, 1300 km southwest and Alice Springs, in the Northern Territory 980 km to the east. The community population is small, approximately 80 residents clinging to their traditional, culturally orientated activities in harsh yet beautiful countryside ([1], pp. 9–11, 28–29).There is a shop, a school, a clinic and an air strip.

Written from an action research/practitioners perspective, this article argues that there is a real need for preventative health and well-being education programs in remote communities in Australia. The results of the integrated, play based, hands on program, conducted in a small remote community school, clearly demonstrate how students became motivated and engaged in their own learning. In addition, anecdotal evidence showed significant evidence of learning outcomes in areas such as health and well-being, English and mathematics. Furthermore, findings of the effectiveness of a Hospital Familiarization Program (HFP) showed a reduction in children’s fear and anxiety of medical procedures. An increase in children’s knowledge of medical equipment was also evident. Moreover, community members became empowered to take a positive and proactive role in their children’s and their own education, promoting physical, social and emotional health.

To facilitate sustainable delivery of this program to a wider audience of students in Australian rural and remote schools, recommendations were made collaboratively with elders from the remote community.

## 2. Cultural Background

Cultural activities such as hunting, traditional secret men’s and women’s business, funerals and major sporting events take precedence over education, and the children are allowed the autonomy of making their own decision about attending school [2]. A death in the community also affects the attendance. The bereaved relatives move out of their home into a “sorry camp” a short distance away from the community and relatives from distant communities may travel to the sorry camp and remain there until after the funeral. For a period of time after the death, that person’s name will no longer be referred to and instead the “no name” *Kunmarnu* will be substituted [3].

Traditionally, Aboriginal children learn by observation and by trial and error. They have their own particular understandings based on their experiences, often within a traditional foundation ([4], pp. 1–14). Aboriginal children also learn their own limits and they are not disciplined until they reach teenage years. Eye contact, particularly between a child and adult is considered disrespectful, and walking away in the middle of a conversation is considered acceptable [3]. These are cultural influences that teachers need to accept and adapt to when working in and with indigenous communities. It is very much a two way learning process ([1], pp. 9–11, 28–29).

### 2.1. The Ngaanyatjarra Language

The majority of people speak Ngaanyatjarra as their first language. Others are speakers of closely-related Western Desert dialects. Ngaanyatjarra has historically only been a verbal language but, in the past decade an increasing amount of written vernacular has been produced in an attempt to retain the language. However, less than 20% of adult Ngaanyatjarra speakers are able to read Ngaanyatjarra, because it has not been taught in schools [5].

Historically, language is the mediator of learning in Western education. However, in the Aboriginal culture and context some people rely on observation and approximations and repeated passing of the unchanged stories and songs about knowledge, wisdom and lore [6]. This practice was clearly an important part of the students’ culture as singing and telling stories in the sand was spontaneously carried out during the school day. The importance of two-way learning was acknowledged and an emphasis placed on socio-dramatic play, storytelling, music, drama and art as an integral part of the health and well-being program.

### 2.2. Community Health Background

The Ngaanyatjarra Lands of 3.1 million sq km have a population of approximately 2500 Indigenous people. The level of poverty in the remote communities has significant implications for health and well-being as it acts as a hindrance to affordability of nutritional food. In addition, the lack of availability of healthy foods is profound and the higher cost in the more remote areas of Australia is significant ([7], pp. 189–218). Fresh fruit and vegetables are transported a long distance and are extremely expensive to buy. For example, the price of one piece of poor quality fruit is around $5 and potatoes cost 50 cents each. According to Burden (2001), this poses a serious problem as many Aboriginal children are failing to thrive ([7], pp. 189–218).

Several communities have no permanent doctor or nursing sister and medical evacuations are not uncommon ([1], pp. 9–11, 28–29). Moreover, the mortality of Indigenous people is 13 times higher than mainstream society and Aboriginal children are rated a one in four chance of developing serious emotional or behavioral difficulties [8]. Chronic Suppurative Otitis Media (CSOM) is a common disease in Aboriginal children [9]. The World Health Organization has indicated that a prevalence rate of CSOM greater than 4% in a defined population of children is indicative of a massive public health problem ([10], pp. 177–178). Alarmingly, CSOM affects up to 10 times this proportion of children in many Aboriginal communities. The associated hearing loss has a life-long impact, as it occurs during the years of speech and language development and the early school years ([10], pp. 177–178). Infectious diseases such as head lice, scabies and tinea (ringworms) are all too common. Some diseases still prevalent in Aboriginal children are virtually non-existent in the non-Aboriginal community, for example rheumatic fever causing significant life-long morbidity [9].

Furthermore, Princess Margaret Hospital for Children, Perth West Australia statistics for 2009 revealed that 13.3% of outpatients, and 17% of inpatients come from rural and remote areas [11]. Timmins (2010) stated that the medical care such as hospitalization and medical treatment may be foreign and frightening for Indigenous people who live in remote communities [12]. They may fear the unknown; being transported thousands of kilometers away from their familiar environment; flying; dying; bad spirits, which many believe reside in hospitals; isolation and lack of family support. Timmins (2010) cites the following case, which clearly illustrates the need for education about hospitalization and medical treatment:

A 17-year old boy from a remote community was evacuated to Alice Springs (980 km away) because of a fractured elbow. I next saw him two weeks later and discovered that on arriving in Alice Springs for the first time ever, became so terrified by this “metropolis” that he immediately set about finding ways to return to his community rather than attend hospital care. He now lives with a permanently dysfunctional elbow.

[13]

In addition, research suggests that for many young children, hospital admission provokes feelings of considerable anxiety. This is particularly so in the case of emergency admission to hospital and treatment by invasive procedures. Adding to the child’s distress, the withdrawal of the child’s familiar routines and surroundings, which are replaced by an environment filled with unfamiliar people, sights, sounds and smells [13]. For some children the ensuing stress may be relatively minor. However, other children have been found to suffer negative psychological effects such as withdrawal, depression, aggression and phobias, sleeping disorders, hyperactivity and erratic control of bodily functions, both during and after hospitalization [14,15,16].

### 2.3. Rationale

To address the poor state of health and the fear of hospitalization, it was decided to implement a three-fold multi-faceted, holistic health and well-being program, featuring the Hospital Familiarization Program (HFP) as a key component [17]. It was concluded that the most important principle would be to ensure that the students and their parents were made to feel welcome and valued; that the school environment was student centered in terms of being interesting, fulfilling, enjoyable, relevant to the culture, safe and empowering.

In 2002 an integrated health and well-being program was developed in consultation with community elders, the Aboriginal and Islander Education Officer, (AIEO) and the local community health worker. In 2008 this program was revised, enhanced and consequently implemented and offered to students and to the local community members [17].

The impetus for this health program development came from the finds of Calmas (2007) who stated that it is our responsibility to improve the social and emotional well-being for our children, families and communities [18]. Consistent with this view, Burden (2001) proposed that if significant improvement is to occur in the health status of Aboriginal people a preventative, holistic approach needs to be adopted ([7], pp. 189–218). Burden recommend that the Department of Education and Training provide significant preventative health programs in schools as part of the health and physical education program and that health programs are developed in conjunction with community members, Aboriginal health workers, clinical staff and allied health agencies. Furthermore, regular meetings should be held between school and local clinic staff to discuss relevant cases and health education within the community context [19]. In addition, this view was supported from 2002–2009 by the Circuit doctors, touring the 11 communities in the Ngaanyatjarra Lands. They stated that the health and well-being program should be part of every remote community school’s curriculum.

## 3. Program Pedagogy

### 3.1. Empowerment

Community involvement in class activities such as art, music and health lessons was encouraged and fostered. Provision was made for informal and formal meetings with parents/caregivers who are acknowledged as first teachers of their children [20] and were therefore encouraged to take a positive and proactive role in their children’s education. The educational task was to enhance the competence of parents/caregivers so that they would use both their resources and their control over family processes to, in turn, enhance the competence of their child.

Health promotion is the process of enabling individuals and communities to increase control over the determinants of health and thereby improve their physical, emotional and community health. It assumes direct involvement of community members in the achievement of change. It also involves political action towards better health for people living in remote communities ([21], p. 187).

Educator Paulo Freire’s (1986) sociological philosophy of empowerment states that the goal of empowerment is not to achieve power and control over others but it aims at giving power to make changes collectively [22]. It is a social action process that promotes participation and dialogue between community members. Empowerment encompasses prevention, self-determination and social justice ([23], pp. 738–739) giving individuals a greater locus of control over their own lives, within their communities and in the wider society. Empowerment education can be an effective health education and prevention model that promotes physical, social and emotional health.

### 3.2. Student Centered Education

It is well known that students who have been part of decision making processes about their own learning, are more likely to feel valued, to be more self-motivated and engaged in any learning process. It is critical, however, that the learning experiences offered are real, concrete and relevant to students, reflecting their world and experiences [24].

**Figure 2:**
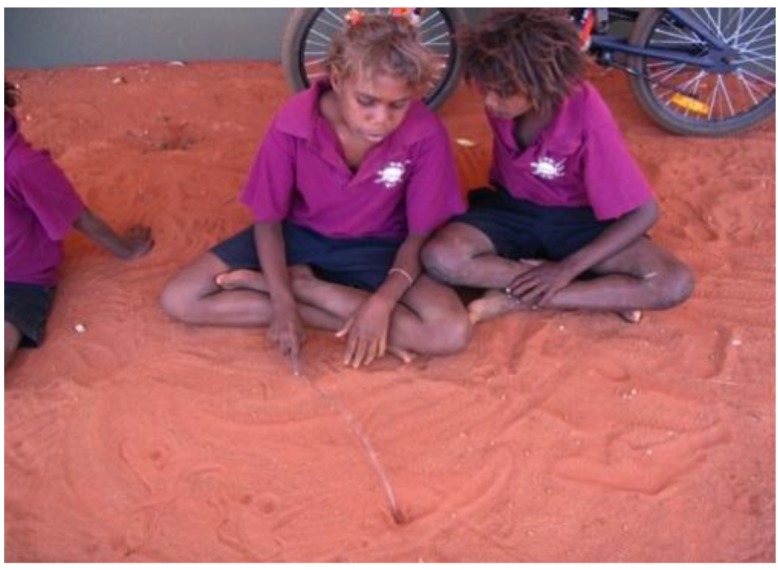
Telling stories in the sand.

Crowther (2005) postulates the significance of a student centered environment with three dimensions: child/adult, structured/directed/explicit teaching and student initiated [25]. Furthermore, students should be challenged through learning experiences that vary in length and complexity, including culturally appropriate methods. Given that Australian Aboriginal children learn by observing and by trial and error [4], the program focused on play-oriented hands on activities. The program included role play about cultural activities such as hunting, music and dancing, hospital play and “telling stories in the sand”, see Figure 2.

### 3.3. Play

Seeing family members airlifted out of the community because of illness was a familiar experience for these children. While some of the children had actually been the patient requiring emergency treatment, for those who had watched a plane carry a community member over the horizon, where the patient was taken and what happened during their absence remained a mystery. In addition to familiarizing children with medical procedures, participation in the HFP helped the children make some sense of what ill community members experienced during their absence ([1], pp. 9–11, 28–29).

Sebastian-Nickell (1992) described play as “one of the most important activities in a young child’s life”, adding that it is “valuable to every aspect of development: physical, social, intellectual and emotional” [26]. Play is a context for learning that enhances children’s thinking so that they become inquisitive, wanting to know and to learn, solve problems and engage in critical thinking. They actively construct their own understanding and contribute to others’ learning. Children become aware of their capacity to initiate and lead learning, and their rights to be part of decision making processes concerning their own learning [27].

Indigenous Elder, Aunty Irene McBride (2008), stated that young Indigenous children should be encouraged to role play more because it is vital to their development as well as to their education [28]. Furthermore, students who have difficulty expressing their feelings and ideas using language are given the opportunity to demonstrate understandings through socio-dramatic play [29]. While engaged in socio-dramatic play children’s ability to self-regulate: to monitor and modify emotions, control impulses, tolerate frustrations, delay gratification and co-regulate in social interactions is significantly enhanced ([30], pp. 899–911).

Through socio-dramatic medical play, children can create their own world, where they are free to act out new roles as they master new situations. Smilansky and Shefatya (1990) point out that the roles taken on by children in play are significant because rather than being the recipients of someone else’s activity, the children are now in control [31]. For example, being the doctor treating the patient, rather than being the patient receiving treatment.

Socio-dramatic medical play also enables children to explore in a non-threatening and safe environment. By exploring unknown medical equipment, such as a drip or a stethoscope they are able to hold, feel, examine and manipulate the object. As a result, a relationship is developed with each object ([32], pp. 687–708), and simultaneously any anxiety associated with the unknown object is reduced [33,34,35].

Gaining control over potentially fearful events may in turn enhance a child’s confidence and feeling of self-worth and hence, the ability to cope better with medical procedures [36,37]. Socio-dramatic medical play may also be therapeutic in the healing process, where children have the opportunity to act out feelings about medical procedures; for example by re-enacting a traumatic event, previously experienced involuntarily ([38], pp. 23–26).

It is important to respect the integrity of socio-dramatic play by protecting the children from adult interference, providing the appropriate space and props, and permitting the children to choose and decide how long they will play [39]; thus, enabling them to come to terms with reality in their own way and in their own time.

Traditionally, Aboriginal children rely heavily on observation and imitation to learn new skills ([4], pp. 1–14). Given that young Aboriginal children living in remote areas of Australia may have limited understanding of the English language, it is important to provide opportunities to develop understanding through engagement in concrete learning experiences, where children can adopt roles and rules that have a culturally appropriate base. As role play has no language barriers, children from diverse language backgrounds will readily participate, acting out what they have learned.

### 3.4. Collaboration

Collaboration with the Aboriginal and Islander Education Officer (AIEO) ensured provision of cultural understanding and appropriate learning experiences. Aboriginal children are used to a higher degree of autonomy at an early stage, and their learning style is based on modeling and trial and error [29]. Acknowledgement that English is the student’s second or third language was inherent in the program’s design and consideration was given to the fact that it is tiring for students to translate and respond to another language [6].

Consistent with the findings of Malcolm *et al.* (1999) and the Deadly Ideas resources [29,32,40] the health and well-being program was underpinned by negotiation, collaborative group work, problem solving, acknowledgement of the students’ home language, integration of curriculum areas and hands-on learning experiences.

Collaboration with doctors, nurses, allied health workers, other related agencies, families/caregivers and specialist teaching staff assisted in achieving better outcomes. This collaboration included frequent informal and formal meetings with all relevant stakeholders.

## 4. The Health and Well-Being Program

**Figure 3:**
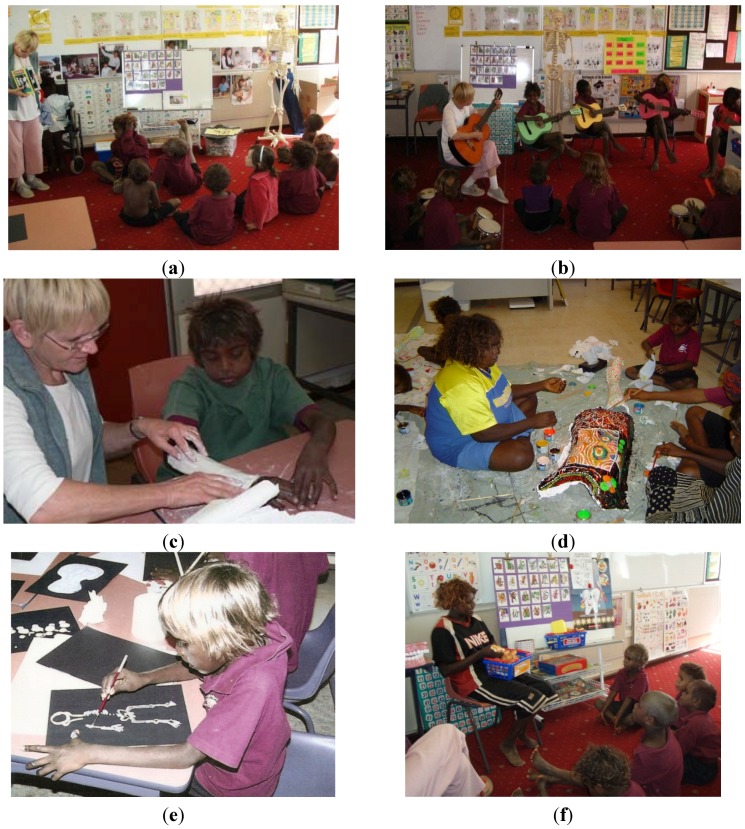
(**a**) Science: Learning about the skeletal system; (**b**) Music: Singing a skeleton song; (**c**) Science and Math’s: Making a plaster cast; (**d**) Art: Painting torsos; (**e**) Art: Painting a skeleton; (**f**) Health: Learning about healthy food groups.

As shown in Figure 3, a to h, the integrated health and well-being program encompassed the curriculum areas of health, science, English, mathematics, technology and enterprise, society and environment and the arts. Its aim was to increase students’ awareness of healthy living; promote healthy self-concept; self-esteem; self-confidence; respect and caring for self; others; the community and the environment. To achieve this, the dual components of physical and emotional well-being were included.

### 4.1. Program Content

Self-management skills, interpersonal skills and basic physiology about the human body, such as the skeletal, muscular, cardiovascular, respiratory, renal, digestive and nervous system were taught. Furthermore, simple research skills and report writing were integral elements of the program, which focused on developing awareness of how to achieve and maintain a healthy lifestyle through personal hygiene, safety, nutrition, sleep and visits to the doctor, dentist and the hospital, The program was differentiated to meet the needs of multiple age groups and individual students’ abilities. Individual learning styles, multiple intelligence and cultural diversity were taken into consideration [17].

### 4.2. Implementation

The daily routine of the health and well-being program began in the morning. On arrival, the students immediately went to the bathroom, where they had a shower and then dressed in freshly-laundered uniforms. This was followed by eating a healthy breakfast; oral hygiene and the breathing blow and cough (BBC) procedures. Healthy morning tea, lunch and filtered boiled water were provided. A 15 min rest or meditation session after lunch was also part of the program.

## 5. Findings

Case studies were made of each individual child and portfolios of the students’ work were compiled, displayed where appropriate, and shared with parents. Following are some snapshots of student’s progress:

In the beginning of 2008, a year two student who had poor listening skills, no knowledge of the alphabet, no phonological awareness, unable to form letters and no knowledge of numbers became a motivated and engaged learner, who accelerated beyond all expectations. By June this student wrote a simple question and answer about the length of the esophagus. In addition, he developed a real passion for reading and mathematics. By the end of the year, the student was able to read more complex text and operate with numbers over a thousand, making additions, subtractions, divisions and multiplications. Another student wrote a simple question and answer about the length of the small and large intestines. Once he had found the answer, he decided to demonstrate to the class what it meant. Using strings he measured the length of the small and large intestines and displayed his findings on the board.

### 5.1. Knowledge of Health Issues

**Figure 4:**
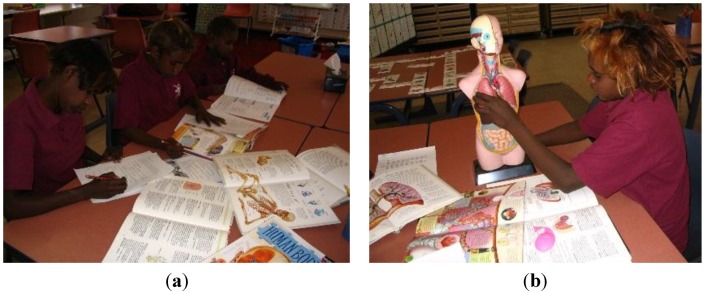
(**a**) English/science: Researching the human body; (**b**) Science: Researching the respiratory system.

A year six student who researched the respiratory system, wrote about having two healthy lungs because she does not smoke. People who smoke, she wrote, have black lungs and if they keep smoking they may develop cancer and die. She stated that she is not going to smoke, because she wants to live for a very long time, see Figure 4, a - b.

**Figure 5:**
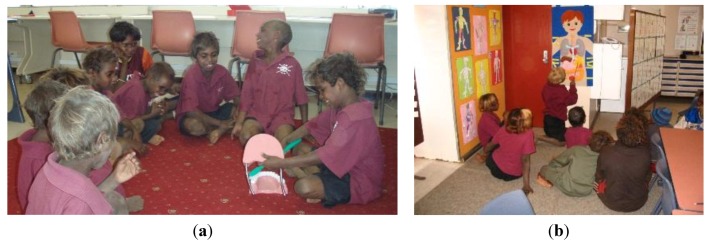
(**a**) Demonstrating oral hygiene; (**b**) Explaining the digestive system.

During a large influx of students from other communities, living in the local sorry camp, one of the year two students decided to demonstrate oral hygiene to the visiting students, while talking about the importance of looking after the teeth, see Figure 5a. This was followed by another year two student who proceeded to demonstrate how the digestive system works, explaining the importance of eating healthy food and keeping the teeth clean. He strongly emphasized how rotten teeth have a bad effect on the digestive system, see Figure 5,b.

### 5.2. Changes of Community Policy

During this time, and in consultation with the advisor and a dietician, local community members decided to develop and implement a new Healthy Store Policy. This included a significant reduction in ordering unhealthy foods for the community store, which contain high levels of salt and fat and drinks, which contain high levels of sugar.

## 6. The Social and Emotional Well-Being Program

The second component of the health and well-being program, designed to support students in developing healthy concepts about themselves as individuals and about themselves as learners, was facilitated through a series of lessons in which the students investigated the brain structure and function, the importance of sleep and exercise; the impact of substances of abuse on the brain; the biology of emotions, with particular emphasis on anger management and critical interplay between emotions and behavior.

**Figure 6:**
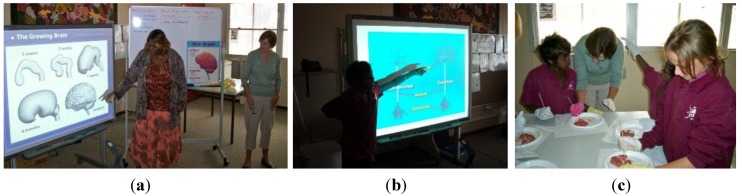
A local community member translating. (**a**): Science: A student explaining; (**b**) Science: Dissecting sheep brains. The anatomy of the brain into the local dialect. (**c**) The anatomy of dendrites.

Informed by neuroscience research and based on the principals of brain compatible learning [41,42,43], this component of the program provided a range of hands-on multi-sensory learning experiences that fostered alternative learning pathways to meet the specific learning needs of these students. Activities included: dissection of sheep’s brains to explore the brain parts that are responsible for balance, coordination and movement; thinking; learning; emotion; relaying information and sleep; construction of model brains and neurons, see Figure 6, a - c creative movement; music; mind mapping and role play.

Students explored the concept of self-talk and rehearsed strategies for taking control of their inner voice and thus maintaining balance between the emotions and the intellect. This was achieved through a range of role play scenarios, through which students explored feelings such as joy, optimism, creativity, loneliness, anger, fear or despair, and developed a vocabulary to describe feelings and emotions. Wright (2006) postulates that through drama, where students take on the roles of others; their social, emotional and intellectual abilities are enhanced [44]. He further reports that role play has been found to improve vocabulary and self-concept.

Role play experiences enable children to develop an understanding that when the emotions are allowed to override the intellect impulsive, volatile behavior is likely to occur. They learned to apply reasoned thought and analysis to situations to avoid habituating to impulsivity and aggression and were encouraged to extend this understanding to the playground and circumstances beyond school. By empowering students with a language to describe feelings, the propensity to react impulsively to situations was clearly minimized.

**Figure 7:**
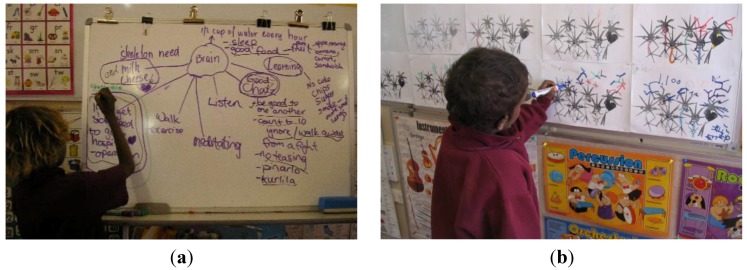
(**a**) Science/English: Making a mind map; (**b**) Recording of new dendrite growth.

Students further created a mind map, see Figure 7 a, and learned about neurons and dendrites and understood that dendritic growth occurs as a result of all new learning. Each student was provided with a “dendrite chart”, see Figure 7, b, on which they recorded new growth when they mastered a new skill. These included academic, social, emotional and intellectual skills.

This segment of the program also dealt with confronting but relevant issues of substance abuse and petrol sniffing. Animated computer graphics were used to guide students’ understanding of the impact of substances on brain function, along with an introduction to the physiology of addiction. Cartoon graphics were used to demonstrate the pathway of substances through the brain to activate the brain’s reward system so that feelings of pleasure experienced in relation to the substance convince the user to want to repeat the experience. Students learned that with each repetition of use of the substance the momentum towards addiction is accelerated.

Students further understood that when a foreign substance is introduced to the brain, the balance between the brain’s chemicals and hormones is upset. This can lead to erratic behavior, loss of cognitive function, loss of appetite, disruption to sleep cycles and breathing.

### 6.1. Findings

Observations of students’ behavior indicated that the social and emotional well-being program was successful. As students internalized the hands-on learning experiences, they became more confident, motivated and aware of their own learning processes. The role play, creative movement and puppetry activities were introduced to support the concept of “self-talk”, providing structures to help them make better choices. They were more confident to resolve issues using dialogue instead of fighting; to listen to others’ viewpoints and to put themselves in the other person’s shoes. There was a significant reduction in aggressive behavior from term one to term four.

Students spoke of new learning in terms of “growing dendrites”. At the end of each day student recorded, on an individual “dendrite chart” provided, how many dendrites they estimated they had grown that day.

## 7. The Hospital Familiarization Program

### 7.1. Introduction

In order to reduce the anxiety of the students about medical intervention and going to hospital, the Hospital Familiarization Program was presented to them. This innovative, play-based program is typically twofold; to inform young children about medical procedures and to teach them effective coping strategies [45]. It has three key elements:

- The first is an interactive group session where children are shown various items of medical equipment and have the opportunity to participate in discussion about their own hospital experiences.
- The second element is the viewing of a DVD in which a child, who has suffered a broken arm, is admitted to hospital and taken through common procedures of hospitalization, including anesthesia and surgery.
- The third, and arguably most significant element, is free socio-dramatic play where children are given the opportunity to dress up as doctors, nurses, surgeons and ambulance drivers in replica, child-sized uniforms. Alternatively, children may elect to play the role of patient or parent.

A wide range of common medical equipment and large persona dolls enable children to further explore the hospitalization scenario through play. In this context, children are able to express their concerns and fears, ask questions and seek assurance from supportive adults as they familiarize themselves with basic items, including drip equipment, bandages, crutches, wheelchairs, stethoscopes, X-rays, plaster casts, blood pressure monitor, thermometer, name bands, books, puzzles and a miniature toy hospital [46].

**Figure 8:**
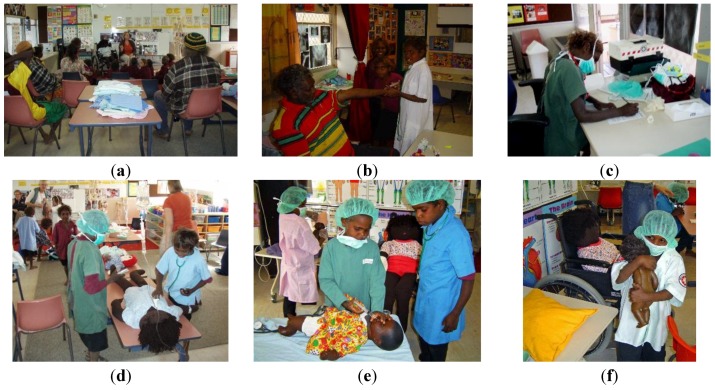
(**a**) The Hospital Familiarisation Program; (**b**) A parent being treated at the “hospital”; (**c**) English: Writing a medical report; (**d**) Doctors treating the patient; (**e**) The consultant and the registrar; (**f**) Caring for the patient.

The pictures below, Figure 8 a – f, illustrate the role-play children eagerly engage in.

### 7.2. Findings

A few weeks after the program, in 2008, a six year old child was evacuated to a major hospital with severe burns to her body. She was accompanied by a family member who stayed with her throughout the hospitalization. In addition to being prepared for hospitalization through the HFP at the school, the Association for the Welfare of children in Hospital (AWCH) supplied the child with a doll, which she used to re-enact the traumatic events of her accident and hospitalization. The family, hospital staff and visiting AWCH committee members, all reported how well she coped with hospitalization. However, more importantly, the little girl, on her return to the community, had brought the doll with her. Using the doll, she was able to communicate her experience to her peers and community members.

Observations of the children engaging in medical play revealed that they internalized what they learned about hospitalization and medical procedures, which in turn will prepare them to face the prospect of medical intervention with confidence and resilience, should such a situation arise.

**Figure 9:**
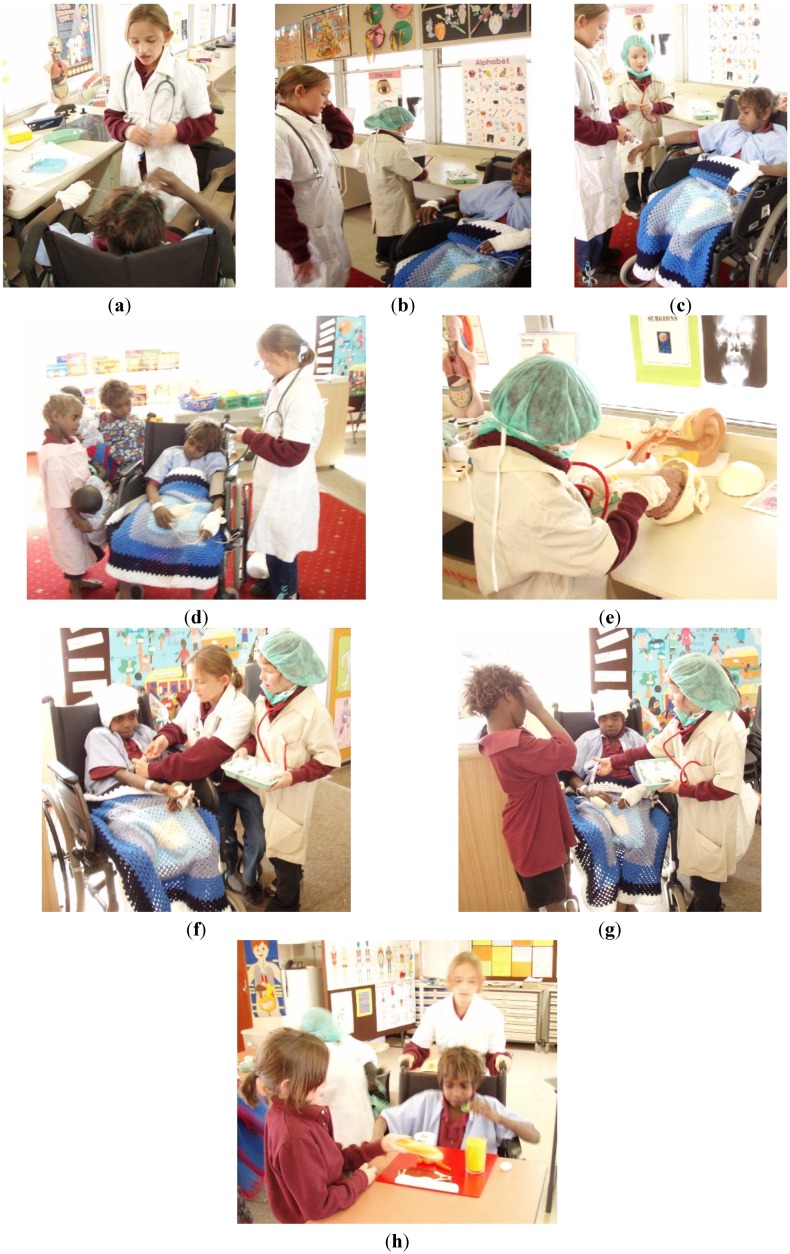
(**a**) The “doctor” tells the patient that he is very sick and that he needs an operation. The “doctor” explains the procedure carefully; assuring the patient that everything is going to be fine; (**b**) “Jack you are very sick and you need an operation on your brain!” “I need to put you to sleep. I will put magic cream on your hand so that the needle won’t hurt you. Before I count to three you will be asleep!”; (**c**) “One–two–three!”; (**d**) “His blood pressure is fine!”; (**e**) The brain surgeon.; (**f**) “The operation went well Jack, everything is fine, but you need more medication so that you don’t get an infection!”; (**g**) “I am sure that your brother will look after you when you get home!”; (**h**) “Make sure you look after your brain Jack. Remember it needs healthy food and half a cup of water every hour. Now eat up all the food and drink the water!”.

Recordings of the children engaging in spontaneous hospital role-play, shown in Figure 9, a to h, clearly demonstrate a sound understanding of the content of the HFP program:

This story clearly testifies the importance of play in helping children cope with trauma. Therapeutic medical play is a healing process, where children have the opportunity to act out feelings about medical procedures as this young girl did, re-enacting a traumatic event, empowering her to release emotional energy and gain mastery over that event.

An evaluation of the effectiveness of the HFP was carried out in 2003 with 16 children aged from five to 16 to ascertain the effect of providing information about common medical equipment and procedures. This was followed by a socio-dramatic play session to test the students understanding of common items of medical equipment and procedures. This was carried out using the Medical Equipment and Procedures Test (MEPT). In addition, the subjects’ feelings towards possible hospitalization and medical intervention were obtained by the use of a Hospital Intervention Feelings Index (HIFI).

An analysis of variance of pre- and post-test measures revealed a significant increase in the children’s understanding of medical equipment and procedures (F = 16.067; d.f.1 and 40; *p* < 0.01). Therefore, the provision of information about medical apparatus and procedures in a developmentally and culturally appropriate manner, followed by socio-dramatic play, resulted in increased knowledge and understanding. In addition, positive feelings towards medical equipment and procedures were found (F = 3.856; d.f.1 and 40; *p* = 0.06) [47].

In other words, the HFP was effective in achieving its intended outcomes of increasing the children’s knowledge and understanding of common items of medical equipment and procedures and of reducing anxiety regarding possible medical intervention [37,47]. Given that the majority of these children suffer from serious health problems and many are prone to accidents requiring hospitalization away from the community, it is important that they receive a program such as the HFP to minimize anxiety and possible trauma due to medical intervention [48].

## 8. Conclusions

Holistic in its approach, and broad in its scope, this highly successful multi-faceted program encompassed the fundamentals of personal hygiene; understandings of body systems; brain care; the importance of nutrition, hydration, sleep and exercise; the impact of substances of abuse on the brain; and the biology of emotions, with particular emphasis on anger management and critical interplay between emotions and behavior.

Collaboration with local community members and health professionals ensured a well-planned, wide-scaled program offering high quality learning experiences for students, community members and staff alike.

The findings clearly show significant outcomes in improved learning and knowledge of health issues. The impact of how cultural consideration consultation and collaboration can lead to prevention and self-determination is also evident.

The process of the health promotion program enabled individuals and the community to become empowered, increasing control over the determinants of their wellbeing and to improve their physical, emotional and community health. As a result of empowerment, community members collectively became directly involved in the achievement of change.

**Figure 10:**
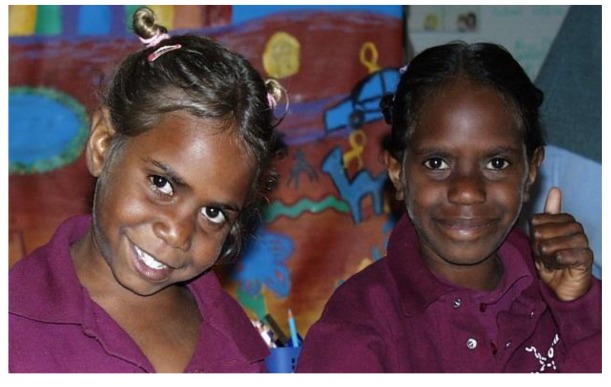
Participants in the program

This program illustrates how empowerment education, through a play based hands on learning environment, can be an effective health education and prevention model that promotes physical, social and emotional health, see Figure 10.

“Tell me and I forget. Teach me and I remember. Involve me and I learn”

—Benjamin Franklin

## 9. Recommendations

1. A DVD be produced featuring an Indigenous child from a remote community going to hospital. The DVD would take the child on a tour of the hospital, taking in the admission procedure, the radiology unit, operating theatre, wards and key hospital personal.
2. In order to ensure sustainability the program should be offered to schools throughout Australia including rural and remote community schools.
3. A program, to be produced, features two main animated sections. 1. Educating children about hospitalization. 2. Educating children about the function of the human body systems, along with attention to how illnesses such as diabetics, renal and cardiac vascular diseases affect the body and how they are treated.
4. Publish the health and well-being model for an international audience, with the view of replicating the program in remote locations around the world.
5. Undertake ongoing research to measure the effectiveness of a health and well-being program.
